# Within-Individual Variation in Cognitive Performance Is Not Noise: Why and How Cognitive Assessments Should Examine Within-Person Performance

**DOI:** 10.3390/jintelligence11060110

**Published:** 2023-06-02

**Authors:** Arabella Charlotte Vaughan, Damian Patrick Birney

**Affiliations:** School of Psychology, University of Sydney, Camperdown, NSW 2006, Australia; arabella.vaughan@sydney.edu.au

**Keywords:** cognitive ability, within-individual variation, psychometric assessment, repeated measures assessment

## Abstract

Despite evidence that it exists, short-term within-individual variability in cognitive performance has largely been ignored as a meaningful component of human cognitive ability. In this article, we build a case for why this within-individual variability should not be viewed as mere measurement error and why it should be construed as a meaningful component of an individual’s cognitive abilities. We argue that in a demanding and rapidly changing modern world, between-individual analysis of single-occasion cognitive test scores does not account for the full range of within-individual cognitive performance variation that is implicated in successful typical cognitive performance. We propose that short-term repeated-measures paradigms (e.g., the experience sampling method (ESM)) be used to develop a process account of why individuals with similar cognitive ability scores differ in their actual performance in typical environments. Finally, we outline considerations for researchers when adapting this paradigm for cognitive assessment and present some initial findings from two studies in our lab that piloted the use of ESM to assess within-individual cognitive performance variation.

## 1. Introduction

Our world is increasingly complex and dynamic. As a society, we face ever more challenging problems, which have a multitude of ethical considerations and resource constraints. In our attempts to meet these challenges as individuals, we are often barraged with information from multiple sources of varying reliability. Yet, despite these progressively more complex and dynamic cognitive demands placed on many facets of our lives, our view of human cognitive abilities remains decidedly static. Attempts have been made to broaden our understanding of intelligence, for example, through dynamic testing and complex problem-solving movements, but progress has apparently not been promising enough to substantially shift the status quo. We argue that what is missing in intelligence research is an understanding of the role of within-individual variability in cognitive performance (Birney and Beckmann 2022).

Variability in cognitive performance has long been recognised as important for understanding the full range of human cognitive abilities (Fiske and Rice 1955). For instance, Spearman (1927) postulated the existence of an oscillation factor that influences the efficiency and accuracy of cognitive performance. Yet, it remains common practice to construct intelligence tests in ways that ensure stability of within-individual differences in the construct they purport to measure. That is, intelligence is assumed to differ between individuals but remain constant within individuals. From this perspective, within-individual variability is an indicator of poor reliability and low validity that should be eliminated during test construction (Birney et al. 2019; Cronbach 1957). Psychometric tests often include practice items, sometimes with feedback, to provide experience with the style of items to be used. This serves to minimise errors in initial items due to construct-extraneous factors that a lack of familiarity with task requirements might introduce. In doing so, this approach further acts to eliminate within-individual differences in cognitive performance and confounds their analysis. Moreover, standard practice ignores a substantial body of research that shows people’s performance on basic cognitive tasks varies even within a short time period (Allaire and Marsiske 2005; Li et al. 2004; Lovden et al. 2007; MacDonald et al. 2003, 2009; Salthouse 2007, 2012; Salthouse and Berish 2005; Salthouse et al. 2006; Schmiedek et al. 2013; Bunce et al. 2004).

Our exposition of the nature and implication of within-individual variation in cognitive ability is presented in three parts. In Part 1, we build a case for why understanding within-individual variability in cognitive performance is important for enhancing our theories of human intelligence and outline a proposed approach for doing so. To date, within-individual variability in cognitive performance has predominantly been conceptualised as noise (Voelkle et al. 2014; Birney et al. 2019) or an indicator of cognitive problems (MacDonald et al. 2009, 2003; Bunce et al. 2004; Lovden et al. 2007). We propose that within-individual variability in cognitive performance can be construed as a meaningful property of an individual’s intelligence and that this is necessary for the development of a process theory of cognitive ability that can provide an explanatory account of why individuals with similar cognitive test results might perform differently in the real world. We argue that the predominantly between-individual approach to the measurement of cognitive ability obscures important within-individual variation in cognitive performance that may have significant implications for the use of cognitive tests (Birney and Beckmann 2022).

In Part 2, we outline methods for examining within-individual variability in cognitive performance. In doing so, we explicate our contention that established paradigms for understanding the magnitude and implications of within-individual variability used in related fields can be adapted to understand within-individual variability in cognitive performance. Specifically, in personality research, within-individual variability has been studied using short-term repeated-measures paradigms, such as the experience sampling method (ESM), which have enriched the field’s understanding of within-person variation as a property of personality that should be assessed independently (Fleeson 2001, 2007; Beckmann et al. 2020).

Finally, in Part 3 we present a case study and some initial insights as to the feasibility of applying the ESM to intelligence testing. We outline some necessary considerations for a cognitive setting and present some initial participant engagement data from two studies in our lab. In presenting this case, we hope to encourage researchers to look beyond the traditional approach to assessing cognitive ability. We argue that embracing a new approach has the potential to enhance both our theoretical understanding of the dynamic nature of human intelligence and our capacity to optimise practical applications of cognitive tests. At the very least, we argue that the extent to which within-individual variation has systematic between-individual differences should be considered more fully.

## 2. Part 1

In the following sections, we present the case for examining within-individual variability in cognitive performance. First, we define within-individual variability in a broad framework of performance variability. Second, we outline the empirical evidence that suggests within-individual variability is a meaningful property of cognitive ability, rather than noise. Finally, we describe why understanding within-individual variability is essential for the interpretation and use of cognitive test results in practical contexts.

### 2.1. What Is Within-Individual Variability?

**Figure 1:**
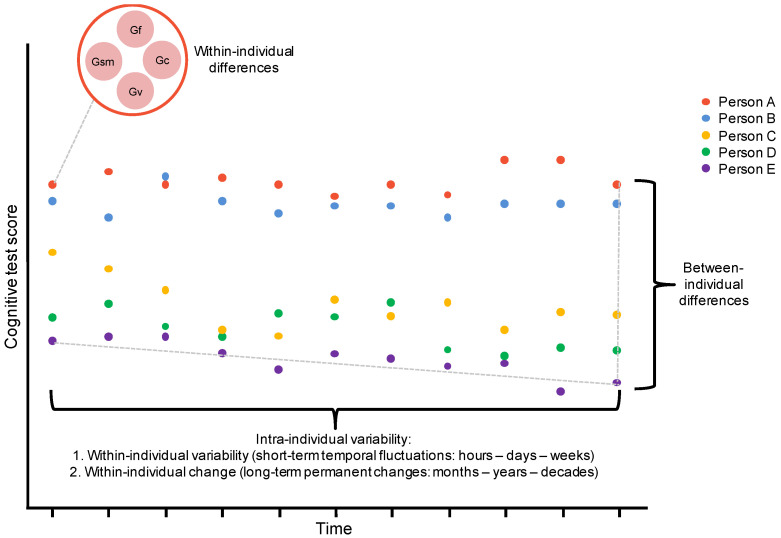
Types of cognitive performance variability outlined in Section 2.1.

We begin by situating within-individual variability in a broader framework for studying cognitive performance variability. Hultsch et al. (2008) distinguish between three related forms of cognitive performance variability, illustrated in Figure 1. Firstly, between-individual differences are the differences between people when measured on a single construct on a single occasion, for example, differences between people’s performance on a general mental ability test. This is the predominant form of variability compared in assessment and selection scenarios. Secondly, within-individual differences are the differences within a person measured on multiple constructs on a single occasion, for example, differences between an individual’s performance on different domains of a general mental ability test, such as Gf, Gc, Gv, etc. Finally, intra-individual variability is the differences within a person measured on a single construct across multiple occasions, for example, differences in an individual’s performance on a Gf test across time.

Nesselroade (1991) distinguishes between two temporal periods of intra-individual variability. Firstly, within-individual change is a form of intra-individual variability whereby an individual’s performance can change slowly and (sometimes) permanently over extended temporal periods, such as years or decades. In Figure 1, within-individual change would be demonstrated if the points on the *x*-axis represented performance over a long temporal period (e.g., years). Secondly, within-individual variability represents fluctuations in an individual’s performance over shorter temporal periods, such as days or weeks. Understanding this variability is closely related to the ESM approach that we will describe shortly and would be represented in Figure 1 if the points on the *x*-axis represented shorter intervals (e.g., days). Of the two, within-individual change has received far more empirical attention through a plethora of longitudinal and cross-sectional research investigating cognitive changes throughout the lifespan, while the attention devoted to shorter-term within-individual variability has been comparatively sparse.

Within-individual variability further encompasses both random and structured variability components. Fiske and Rice (1955) distinguish between Type 1 variability, which is spontaneous or random variability, and Type 2 variability, which is reactive or structured variability. When considering within-person variation in cognitive performance, it is structured variability that we are interested in studying as doing so may help tells us how and why people systematically vary in their typical intellect-related behaviour. This structured variability can occur both across and within measurement occasions (as detailed in Section 3.2.1). Cognitive performance variation is likely to be structured in regard to a variety of factors; in this article we focus on changes in situation, task, and person-related factors (as detailed in later sections).

### 2.2. What Is the Evidence for Within-Individual Variability?

We now turn to the question of whether there is evidence for within-individual variability in cognitive performance. Empirical evidence suggests that people demonstrate both within-individual change and within-individual variability on cognitive tests. Firstly, within-individual change is evidenced as both the scores and factor structure of cognitive abilities are temporally unstable (Tucker-Drob 2009; McArdle et al. 2002). Different cognitive abilities show consistent age-related changes; fluid intelligence (Gf) and speed-related abilities show initial increases and then pronounced declines throughout the lifespan, whereas crystallised intelligence (Gc) and auditory/visual processing plateau and decline at a far shallower rate (McArdle et al. 2002). Furthermore, the first factor extracted from large cognitive test batteries, the *g* factor, accounts for more or less variance in cognitive performance depending on both age and baseline cognitive ability (Tucker-Drob 2009). It too (i.e., *g*) is also considered to undergo qualitative change developmentally over time (Demetriou et al. 2023).

There is also substantial empirical evidence that suggests within-individual variability is an important aspect of cognitive performance, although most of the extant research has focused on basic cognitive processes, such as reaction time (see, e.g., Li et al. 2004; Lovden et al. 2007; MacDonald et al. 2003, 2009; Salthouse and Berish 2005; Bunce et al. 2004; Hultsch et al. 2008). Salthouse and colleagues have demonstrated that within-individual variability in cognitive performance is large on various tasks. For reaction time measures, it is roughly of the same proportion as between-person variability (see (Salthouse and Berish 2005) who conduct an ESM-style study similar to what we will do in Part 3). On more complex cognitive tasks (e.g., vocabulary, inductive reasoning, spatial visualisation, episodic memory, and perceptual speed) the median ratio of within-person variability to between-person variability was 0.54 (ranging from 0.28 to 0.78) (Salthouse 2007). Allaire and Marsiske (2005) found that across a 60-day testing period people displayed substantial within-individual variability in performance and that variability in one domain was generally unrelated to variability in another domain. Moreover, higher performance variability was associated with higher mean performance in a given domain (*r* = 0.34 for perceptual speed and *r* = 0.43 for working memory). Similarly, Salthouse et al. (2006) found substantial within-individual variability in cognitive test performance that varied in magnitude between individuals across a 2- to 10-week period (the median ratio of within to between-person variation was 0.46). In a follow-up study, Salthouse (2012) reported that individuals exhibited between-session variability equivalent to one or more decades of cross-sectional age-related performance differences (with a mean between-session variability of 8.8 years of age-related performance differences), suggesting people vary substantially in their performance in relatively short temporal frames.

In sum, the extant literature suggests that despite the best efforts of cognitive test developers to weed out within-individual sources of variation in test development, people do vary in their performance on cognitive assessments across relatively short time periods. That is, there is indeed evidence for systematic within-individual variability in cognitive performance. Further, this variation cannot be attributed to cognitive decline or maladaptive cognitive functioning because it has been replicated in younger populations without clinically significant cognitive functioning impairments (e.g., in Salthouse 2007; Salthouse et al. 2006); however, the magnitude and nature of this within-individual variability has not been well integrated into our theories of human intelligence. In fact, outside of motivation as an explanatory account, little is known of the sources of within-individual variability in higher-level cognitive abilities.

### 2.3. Why Is Within-Individual Variability Important?

If an understanding of within-individual variability is not integrated into our theories of human intelligence, there is and will continue to be a disconnect between the predominant theory and methodology for operationalising and measuring cognitive ability and the actual nature of cognitive abilities in the real world. There are a number of reasons for this. First, consider a typical cognitive assessment situation. A major national bank is recruiting for their graduate program. As a desirable workplace, they have thousands of applicants to assess and rank, most of whom have impressive academic records. The bank invites several hundred candidates to complete an online cognitive assessment. On the basis of these test results, as well as interviews and reference checks, the bank selects the highest performing students for its graduate program. Yet, several months into the graduate program, it is apparent that the actual workplace performance of the graduates varies substantially—both between and within the individual graduates. All came in with stellar records and all achieved well on the cognitive assessment, so why?

At present, theoretical and practical research into intelligence predominantly relies on such between-individual comparisons of single-occasion scores, or rather, it is often framed in that way (that is, to compare individuals and to select one person over another). Between-individual comparisons of test scores obtained at a single point in time in a controlled environment cannot well-capture those aspects of cognitive ability that vary within individuals. The test situation facilitates maximum concentration and cognitive engagement, either through the test being taken in a testing centre or by the test taker selecting an optimal physical location to take the test. The content and design of the task also demands a narrow form of cognition; cognitive assessments are generally static tasks comprised of a series of independent items, with no elements that change dynamically or based on previous test taker responses^1^. Mostly, only multiple-choice response options with a binary correct/incorrect outcome are available. Further, motivation is usually at its peak because something valued is at stake (e.g., a job or a spot on an educational/training program), so the situational contexts are often similarly narrow.

This approach is extremely different to the actual cognitively challenging situations we encounter on a day-to-day basis. Moreover, it ignores the empirical evidence that individuals can exhibit very different patterns of within-individual variability in cognitive performance across relatively short time periods. Successful real-world cognitive performance requires dealing with dynamic problems, which necessitates the capacity to be sensitive to changes in the problem as well as to changes in ones’ goals over time, flexibility to adapt to these changes, and the capacity to integrate new information into existing schemas. Even with recognition that individual differences in motivation and engagement will fluctuate, dynamic changes in our environment demand a cognitive capacity unlikely to be captured sufficiently in static intelligence tests, which have been designed to be sensitive to between-person differences. While some have proposed different constructs, such as practical intelligence (e.g., Sternberg et al. 2000), to deal with contextual variability, our focus here remains on the set of classic cognitive abilities; however, we frame these classic cognitive abilities from a within-person perspective described as short-term temporal fluctuations in Figure 1 rather than as only between-individual differences.

**Figure 2:**
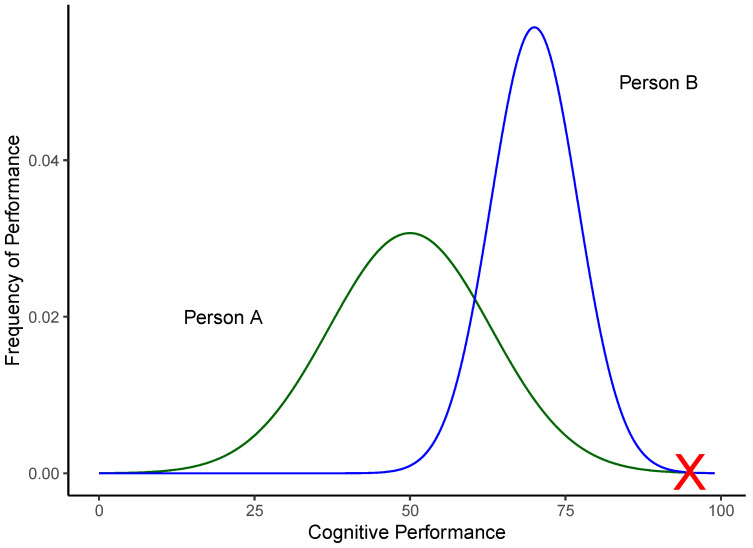
Individuals with the same maximal performance and different typical performance.

Understanding real-world cognitive performance therefore requires an understanding of structured within-individual variability in response to changes; in other words, we need to embrace heterogeneity (Bryan et al. 2021). This is not to the exclusion of between-individual comparisons. Rather, it is intended to complement and enhance the utility of between-individual comparisons by providing a within-person process account of between-person differences in cognitive ability, much in the same way as nuanced personality *states* have augmented our understanding of personality *traits*. As an example, consider two individuals who achieve similar results on a cognitive assessment when a place on a competitive graduate program is at stake, as represented by the red X in Figure 2. Despite their similar peak sore, the individuals exhibit different patterns of within-individual variability in other situations as represented by different distribution curves. This between-person variability in within-person variability (distributions) may be structured in regard to situation, task, or personal factors (e.g., one individual may learn skills and concepts faster than the other or be more sensitive to changes in the problem space and thus quicker to incorporate these into their problem-solving strategy).

While Figure 2 is purely illustrative, the intention is to draw attention to the fact that single-occasion tests taken in conditions designed to minimise within-person variability do not provide a holistic understanding of how or why someone is likely to dynamically vary in their performance across days, weeks, months, or even several years. If we are selecting for a role where consistent high performance is important and mistakes are costly, such as a surgeon or pilot, most people would prefer one whose performance is of consistently high quality and less prone to variation, rather than one whose performance varies substantially. It is for such reasons we suggest that understanding within-individual cognitive performance variability is crucial to holistically understand the cognitive abilities underlying human intelligence, design appropriate cognitive tests, and to interpret results appropriately to make an optimal decision.

## 3. Part 2: Methods for Examining Within-Individual Variability in Cognitive Performance

In Part 2, we review how related fields have incorporated an understanding of within-individual variability into theories and measures, particularly the experience sampling method (ESM) and discuss implications for cognitive assessment (Section 3.1). We then outline the considerations for researchers when adapting the ESM paradigm to capture short-term within-individual variability in assessed cognitive ability. We focus on theoretical (Section 3.2), methodological (Section 3.3), and analytical considerations (Section 3.4). In doing so, we compare existing methods for examining within-individual variability in personality with our proposed approach for examining the same in cognitive ability.

### 3.1. How Do Related Fields Capture Within-Individual Variability?

Within-individual variability has been well integrated into theories and methods for assessing a range of psychological constructs, notably affect, emotion, and personality (Ilies and Judge 2002; Conner et al. 2009; Trull et al. 2015; Beckmann et al. 2020). Importantly, all of these fields have gleaned an understanding of the role of within-individual variability by pivoting away from the predominant use of single-occasion between-individual assessments and comparisons. In particular, within-individual repeated-measures research designs have allowed researchers to uncover how and why individuals vary in their experiences across a typical week or month (Ilies and Judge 2002; Conner et al. 2009; Trull et al. 2015; Beckmann et al. 2020). Generally, this research leverages the experience sampling method (ESM).^2^ The ESM takes many short measures (over a week, fortnight, or month) and is based on a simple premise; taking a sample of people’s experiences over a short time period allows us to more deeply understand the variability in their thoughts, feelings, behaviours, and experiences as they unfold in their everyday lives (Conner et al. 2009). The spreading of measurement occasions across several weeks allows researchers to sample fluctuating experiences across a range of contexts and to understand how variability is related to various outcomes (Conner et al. 2009; Fisher and To 2012).

In personality research, the ESM has allowed researchers to study within-individual variation in personality, which has resoundingly challenged many of the central tenets of nomothetic personality theory. Importantly, these findings have complemented between-individual comparisons by providing a richer process account of how and why individuals differ in their personality expression over time. This idiographic approach has notably been explored by Fleeson and colleagues (Fleeson 2001, 2007; Fleeson and Jayawickreme 2015), who demonstrated distinctive within-individual personality profiles using the Big Five personality traits. Across multiple studies, Fleeson asked participants to complete a measure of the Big Five several times a day for several weeks in a row. Fleeson found that people exhibited relatively low consistency in self-reported personality states. Rather, there was more variability within individuals than between individuals. As a result of these findings, he proposed that personality is better conceptualised as a density distribution of states that is unique to an individual, with each state more or less likely to be expressed depending on situational cues (e.g., if the situation is a novel social environment, then personality state is more/less extraverted). More recent research has sought to understand the factors that systematically influence within-person variability in personality state expression and has uncovered a range of situation and person factors implicated in the dynamic nature of personality (Beckmann et al. 2021, 2020; Wood et al. 2019).

We propose that the ESM and accompanying within-individual analysis techniques be used to integrate an understanding of within-individual variability in cognitive *performance* into our theories of and methods for assessing cognitive *ability* (and intelligence in general). It is necessary to obtain multiple measures of individuals’ cognitive ability over short time periods under structured conditions so as to better understand how and why they vary in their cognitive performance. We do not propose such a paradigm be used to the exclusion of traditional, well-validated methods for assessment and analysis. Instead, we believe that the findings in personality research are indicative of the potential to also enhance our theoretical understanding of the extent and magnitude of within-individual cognitive performance variability, which can be integrated into existing theories of intelligence and methods for cognitive assessment.

### 3.2. Theoretical Considerations

The theoretical rationale for studying within-person variability in cognitive ability will inform the way in which short-term repeated measures paradigms are designed. In this section we compare how within-individual variability in personality is conceptualised relative to how within-individual variability in cognition is conceptualised, as well as how personality and cognitive states and traits are similar and different. We then consider the implications of this for specifying the structure of within-individual variation and task choice.

#### 3.2.1. Conceptualisation of Variability

In Section 2.1, we situated within-individual variability in a framework of cognitive performance. We outlined two forms of within-individual variability described by Fiske and Rice (1955)—Type 1 (spontaneous or random variability) and Type 2 (structured variability)—and emphasised that it is the latter that we are interested in understanding in this line of research. We now elaborate on why structured variability is of interest when studying personality and cognitive ability.

For personality, variability is construed as a systematic and structured response to situational cues (Fleeson 2001, 2007). Personality state expression is primarily determined by an individual’s distinct pattern of if-then contingencies, e.g., if the social context is familiar, then act more extraverted, but if the social context is unfamiliar, then act less extraverted (Fleeson 2001, 2007). Empirical evidence would suggest that the same is likely to be true of cognitive performance; within-individual variability may be a systematic response to changes in situation, task, and person factors. Cognitive performance can be construed as having two layers of structured variability: between-occasion variability and within-occasion variability.

*Between-occasion variability* entails structured differences in response to situation, person, and task factors that change across measurement occasions. The role of a situation, in which a task is completed, has been explored extensively in the theory of maximal and typical performance. In high stakes situations with high perceived importance, people are expected to aspire for maximal performance (von Stumm et al. 2011; Goff and Ackerman 1992). Variability is primarily dictated by one’s cognitive ability because non-cognitive factors, such as motivation and engagement, are mostly constant across individuals, thus performance is at its peak. As the stakes and perceived importance of the situation decrease, people display more typical performance (von Stumm et al. 2011; Goff and Ackerman 1992). Typical performance is dictated by variation in both cognitive ability and non-cognitive factors, and between-occasion differences in situation valence are likely to interact with task demands. More discretionary effort and motivation is required to voluntarily engage greater cognitive resources in lower-stakes activities because the expectancy of a valued outcome is relatively lower (Wigfield 1994). Furthermore, person factors, including motivational and conative dispositions, such as conscientiousness, openness/intellect, need for cognition, and goal orientation, have been shown to incrementally predict typical performance beyond cognitive ability (Schmidt and Hunter 1998; Schmidt et al. 2016; Strobel et al. 2019; Steinmayr et al. 2011). Finally, differences in task demands and design are likely to contribute to between-occasion variability as the tasks presented to us change across measurement occasions.

*Within-occasion variability* primarily entails structured differences in task factors that change while one attempts the task. Changes in the impact of situation and person factors are potentially an additional source of within-occasion performance variability. For example, in both cognitive tests and real-world cognitive challenges there may be changes in the content and design of the task, and the framing of the situation while the task is underway that affects ongoing task performance. Within-occasion variability may also be impacted by some of the aforementioned person factors, which affect engagement with cognitive challenges and responses to changes in the task or situation. Analysing within-occasion variability is important because it reveals information about people’s response to change, their strategy use, cognitive exploration, and flexibility (Schmiedek et al. 2010). These critical aspects of cognitive ability cannot be revealed by between-occasion analysis alone. Variability in cognitive performance represents something both similar and distinct from variability observed in personality research, which necessitates the analysis of different layers of structured within-individual variability.

#### 3.2.2. Conceptualisation of States and Traits

Theoretically there are also similarities and differences in the conceptualisation of states and traits for personality and cognition that are important to consider when adapting the ESM paradigm to assess within-individual variation in cognitive performance. In personality research, personality traits are assumed to reflect an average level of behaviour across time (Fleeson and Jayawickreme 2015). This is quantified based on an individual’s self-reflection at a single time point on their usual behaviour or, in the case of repeated-measures studies, based on the average of assessments across time. Conversely in cognitive ability research, the ability traits are generally thought to reflect the maximum level of behaviour, which is quantified as a score (usually) observed in a high stakes situation at a single time point (von Stumm et al. 2011). Consequently, trait personality and “trait” cognitive ability are theoretically different; trait personality reflects typical behaviour, whereas “trait” cognitive ability reflects maximum behaviour.

Both personality and cognitive states share similarities in their conceptualisations. The expression of personality and cognitive states is based on a conscious or subconscious judgment of the environment and a conscious or subconscious decision to allocate resources to navigating the situation (Fleeson 2007; Wigfield 1994). When these states are measured on multiple occasions in personality research, a distribution of within-person variation in states is observed (Fleeson and Jayawickreme 2015). This distribution shows both the trait and its nature of within-person variation (e.g., range, shape, skew) and can be used to understand the factors that influence the expression of a particular state (Fleeson and Jayawickreme 2015). Moreover, the degree of adaptability in personality or cognitive state expression is likely to be linked to different outcomes (Ram and Gerstorf 2009), such as in the workplace or education. In other words, both the average trait level, and the shape of one’s personality and cognitive performance distribution are likely to be reflected in successful life outcomes, albeit in different ways.

However, the conceptualisations of personality and cognitive states differ in an important way. Personality states are captured in a relatively simple momentary reflection on one’s current feelings. This is because personality states are assumed to unfold organically and subconsciously; we do not have to exert substantial effort to answer personality survey items because our personality state is generally not something we consciously influence. On the other hand, our theoretical conceptualisation of successful cognitive performance in demanding environments is of one’s capacity to consciously engage and respond to discrete cognitive challenges, and to changes in these challenge once we are engaging with it (e.g., at school, university, or the workplace) (Birney and Beckmann 2022). To respond to these cognitive challenges, one must effortfully disengage from other tasks, reflect on the challenge presented, and deliberately and effortfully allocate resources to complete it. Faithfully capturing a cognitive state, whether using an ESM paradigm or not, requires longer and more engagement-demanding measures than capturing personality states, and this has implications for the type of task used.

#### 3.2.3. Structure of Variation

The theoretical conceptualisation of variability, states, and traits has implications for the likely structure of cognitive ability variation, and this has ramifications for the adaptation of ESM paradigms to cognitive assessment. At its core, expression of momentary personality and cognitive ability states involves the allocation of our finite cognitive, behavioural, and emotional resources based on some appraisal of the situation. This allocation of resources represents a structured and systematic response to situation, task, and person factors at the time the measurement is taken. In personality research, situation factors have increasingly been shown to systematically influence within-individual variation, for instance, by the degree of familiarity of those we interact with, whether it is a social or workplace context, and the particular reason for the social interaction (Rauthmann et al. 2016, 2015; Sherman et al. 2015; Beckmann et al. 2020).

However, as we have outlined in Section 3.2.1, cognitive performance is structured between tasks and within tasks. Moreover, as described in Section 3.2.2, the complex nature of cognitive task performance means that it is likely to be interactively structured in respect to situation, person, and task factors; therefore, when developing an ESM paradigm to assess structured within-individual variability in cognitive performance, we will likely need to capture more sources of structured variability than in personality research. As a starting point for further research, it is instructive to briefly outline some situation, person, and task factors that may systematically influence cognitive task performance.

*Situation factors* that influence performance may include the stakes of the situation in which a task is completed (von Stumm et al. 2011; Goff and Ackerman 1992). In personality and affective dynamics research, measures have been established to evaluate situation-contingent responding. One popular measure is the DIAMONDS scale (Rauthmann et al. 2014, 2016, 2015), which captures eight situational features that may influence behaviour: duty, intellect, adversity, mating, positivity, negativity, deception, and sociality. The empirical evidence for the interaction between DIAMONDS and personality is limited (Sherman et al. 2015; Rauthmann et al. 2016); however, this may be due to the similarity of content captured in DIAMONDS and other personality measures, e.g., sociality bears strong similarities to agreeableness and extraversion, and intellect bears strong similarities to openness/intellect. For the study of cognitive ability, DIAMONDS, or something similar, may be useful to understand situational influences. As there is little, if any, construct overlap between these situation characteristics with cognitive ability, DIAMONDS ratings may explain unaccounted for variance in cognitive states. Equally, however, the factors may simply be too dissimilar to those that influence cognitive performance. In either case, there is research to be conducted to better outline the situational features that systematically influence within-individual variation in cognitive performance.

*Task factors* include task design and complexity,^3^ the cognitive load of the task (including the presence of intrinsic, extraneous, and germane cognitive load) (Beckmann 2010), and the introduction of new features or information into the problem space (Goff and Ackerman 1992). In addition to the specific cognitive ability being assessed, *person factors* include the aforementioned individual differences traits, including conscientiousness, openness/intellect, need for cognition, and goal orientation, which predict typical cognitive performance (Schmidt and Hunter 1998; Schmidt et al. 2016; Strobel et al. 2019; Steinmayr et al. 2011). Other more transient person factors include skill acquisition, neuromodulatory processes, stress, and motivation (Schmiedek et al. 2010).

Importantly, these situation, task, and person factors are likely to interactively determine patterns of within-individual variability in cognitive performance. For example, individuals may respond differently to changes in the stakes of the situation depending on their motivation, which may in turn be influenced by the complexity of the task presented to them. A key strength of using the ESM to understand within-individual variability is that by holding certain factors constant, we can elicit how people respond to changes in other factors over repeated administrations. To elaborate on the previous example, we could hold the stakes of the situation constant (e.g., high stakes), manipulate task complexity, and ask participants to provide indications of their motivation and engagement over multiple occasions across time. Thus, what we would capture and isolate would be both motivational and task performance changes in response to changes in task complexity under a specific situation. Consequently, a critical aspect of using ESM paradigms to capture within-individual variability in cognitive ability will be the manipulation of one or more situation, task, or person factors while holding other factors constant so as to elucidate the unique and interactive contribution of various factors to performance differences.

#### 3.2.4. Task Choice

If our aim is to understand structured within-individual variation in cognitive performance, we must employ tasks that enable observation and analysis of variability in the processes people typically use to solve a cognitive challenge. Typical every-day cognitive tasks are dynamic and complex, and require motivation and engagement to learn, apply, and master skills and concepts across different activities and contexts (Goff and Ackerman 1992; von Stumm et al. 2011). A static task, such as a standard reasoning measure employing only one type of test item (e.g., all figural matrices), is unlikely to replicate a typical environment because it does not require dynamic processes and this type of engagement diversity. Moreover, single-item or very short cognitive tests do not allow for the extraction of metrics that are important for understanding typical ability processes, such as learning trajectories, cognitive exploration, and complex problem-solving (Birney et al. 2019). How rapidly people learn and apply new concepts, and their cognitive exploration styles has theoretically stronger links to typical cognitive abilities than performance on a binary score on a single item.

Consistent with Birney and Beckmann (2022), it is for this reason that we propose that dynamic cognitive tasks are by definition the most appropriate tasks to elicit meaningful within-individual variability. Specifically, these tasks allow analysis of different levels of time-structured variability—between-occasion variation (i.e., performance trajectories across different measurement occasions) and within-occasion variation (i.e., performance trajectories within measurement occasions, across different trials), both of which were outline in Section 3.2.1. These trajectories allow researchers to understand factors that influence learning and insight in typical cognitive performance environments, and may be modelled in, for instance, complex problem-solving tasks and microworlds. Other interesting tasks features that may be susceptible to manipulation (and therefore investigation) include insight problems (for understanding dynamic aspects of creative cognition) and strategy tasks (for understanding strategy selection and application in cognitively challenging scenarios).

### 3.3. Methodological Considerations

There are also methodological considerations when designing an ESM style cognitive assessment. Here, we focus on several key method and design issues: the appropriate duration and spacing of repeated measures assessments, and considerations for participant recruitment and retention.

#### 3.3.1. Duration and Spacing of Measurements

To summarise Section 3.2, within-individual variability in cognitive performance is likely to be structured between and within measurement occasions, and influenced by situation, task, and person factors. It is consequently a complex phenomenon and significant consideration should be given to task choice, as well as the choice of situation, task, and person variables to manipulate for investigation and assessment. When analysing within-person variation, it is also critical to align the duration and spacing of measurements with a theoretical conception of the phenomena being measured (Ram and Gerstorf 2009). Within-individual variability in personality state expression is a phenomenon that usually unfolds below the threshold of conscious awareness and thus likely requires participants to merely reflect or report on their current state. Consequently, personality states have been relatively well measured with one or only a few items per trait that collectively take one or two minutes to complete (Rauthmann et al. 2015, 2016; Sherman et al. 2015). Accordingly, personality measures are relatively unobtrusive, can be completed in a minute or two, and do not require a substantial shift in focus from whatever the participant was engaged with prior to the prompt to respond (Fisher and To 2012). As a result, it is feasible to have several measurement occasions per day (Jones et al. 2017; Church et al. 2013; Fleeson 2007). This allows researchers to capture transient changes in personality state expression triggered by fluctuations in the external environment experienced during a typical day and then to investigate the structure of this variation (Rauthmann et al. 2016, 2015; Sherman et al. 2015).

Conversely, within-individual variability in cognitive ability states may be more enduring within a day, and thus, the factors linked to structured variability may sometimes persist for longer, for example, due to cognitive fatigue or motivational factors, such as workplace engagement and perceived task importance. Moreover, as outlined in Section 3.2.4, regardless of whether a static or dynamic task is chosen, ideally it will need to be of sufficient length to capture between- and within-occasion variation. Consequently, ESM cognitive studies are likely to present participants with longer tasks than is standard in personality studies. In addition, cognitive tasks require a significant shift in attention and motivational resources away from whatever the participant was previously doing. This will be more taxing and arguably necessitate greater participant commitment and engagement than for personality studies. Accordingly, participants may need to be given longer time windows to complete a cognitive task after receiving an ESM prompt, e.g., several hours rather than an hour or less as in personality research.

When studying cognitive ability variation, we may also only be interested in understanding variation of experience across a constrained time period. For example, if our goal is to assess ability variation as an indicator of future workplace performance, we might focus measurement occasions during usual working hours, rather than throughout a whole day as is commonplace in personality variation studies. Taken together, this suggests that cognitive studies with repeated measures will need to be limited to fewer but longer measurement occasion/response windows; that is, rather than five 1 min tasks per day we might administer one 5 min task per day.

#### 3.3.2. Participant Recruitment and Retention

As we have outlined, short-term repeated measures cognitive studies are likely to be substantially more demanding than short-term repeated measures personality studies. As a result, strategies to boost participant recruitment and retention should be considered. Participant remuneration may need to be altered, for example, paying above standard rates. Researchers may also want to consider awarding bonuses to those who complete a certain number of tasks to incentivise high completion rates. In student samples where remuneration is in the form of course credit, there may be a benefit to setting high completion rate thresholds so that there are more data points per participant, which increases measurement reliability and statistical power.

Another method for incentivising participant retention is task design. Gamification of the task environment via engaging semantics, animations, and interface design may assist to boost participant engagement. This could be done by embedding tasks within a relevant context and using interactive elements (task feedback). Such manipulations are entirely consistent with a goal to use more dynamic rather than static tasks (Birney and Beckmann 2022).

### 3.4. Analytical Considerations

Once theoretical and methodological considerations have been factored into assessment design, there are several analytical considerations that will inform the data cleaning and analysis approach. Here, we consider how data pre-processing and cleaning could be approached. We also present considerations for extracting ability parameters from data, whether ability is construed as performance scores or response time, and introduce the implications of these parameters for reliability and boundary effects within the data.

#### 3.4.1. Data Pre-Processing

Because cognitive tasks take longer to complete and are more intrusive on participants’ time, researchers may need to be more circumspect when pre-processing repeated measures cognitive data. In repeated measures personality studies it is common to exclude tasks completed more than an hour after it is sent to participants (Church et al. 2013; Fisher and To 2012; Jones et al. 2017; Rauthmann et al. 2015, 2016; Sherman et al. 2015). As outlined above, this approach may be unfeasible in cognitive ESM studies. Furthermore, exclusion criteria for purportedly non-serious responding may need consideration. Excluding based on extreme performance scores or response times may lead to an unrepresentative sample of data variability, particularly if the cognitive task is dynamic. Dynamic tasks present participants with the opportunity to explore a novel system and, as a result, extreme scores may reflect exploration for some, and non-serious responding for others. Including extreme scores in data analysis may tell us something important about people’s typical cognitive ability variability. The fact that the distribution of a person’s performance variation includes extreme exploration strategies and/or non-serious attempts may be an indicator of a substantive cognitive strategy when presented with challenges. In turn, this may be important for understanding applications of cognitive abilities in educational and occupational contexts. In sum, we suggest that researchers develop their data cleaning approach in consideration of faithfully replicating the phenomena of interest rather than simply adhering to conventional exclusion criteria.

#### 3.4.2. Extracting Parameters from Data

Which parameters to extract from cognitive ESM data is an open question and likely requires further theoretical consideration and experimentation. Personality state items are restricted in their response options. Generally, participants are asked to indicate how well an item describes their current state. The variability in these ratings across time can then be analysed using a range of metrics. Common metrics include individual standard deviation (iSD), variance, absolute range, interquartile range, and mean squared successive differences (MSSD) (Ram and Gerstorf 2009; Wendt et al. 2020). These metrics all provide an indication of how much an individual varies across time. More recently, multilevel modelling methods have been employed to understand how personality states vary contingent on other factors over time (see, e.g., Wood et al. 2019; Beckmann et al. 2020, 2021). Regardless of the modelling method used, there is an emerging consensus that one’s current personality state can be captured by a single, point-in-time score. How we select an outcome variable from a cognitive ability task intended to capture within-individual variability is not as clear.

**Figure 3:**
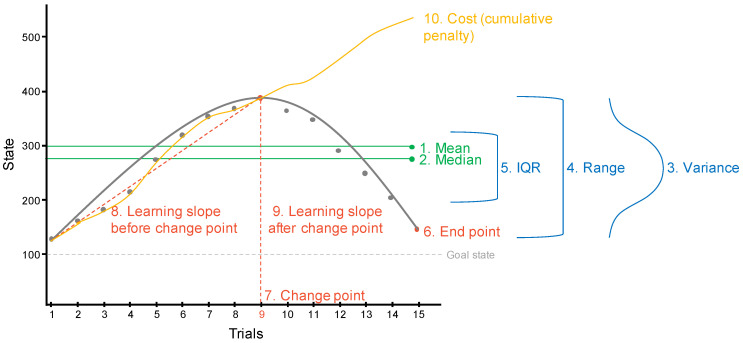
Example of performance metrics extracted from a dynamic microworlds task.

To explicate the challenge, consider performance on a typical dynamic inventory-management style microworld task (e.g., Birney et al. 2018), as represented in Figure 3. Here, the participant is asked to maintain a system state of 100 and given 15 trials to do so. There are numerous possibilities for parameterising the state of the system, some are illustrated in Figure 3. As for personality data, we could take a simple metric, such as the mean or median state, the variance, range, or interquartile range of states, the end point (final trial state), or the cost (cumulative state penalty of all decisions), shown in Figure 3 points 1–6 and 10. These metrics tell us something about someone’s mean level of performance and variability, as well as parameterising the amount of exploration of the system; however, these metrics likely miss fundamental structural aspects of within-individual variability in cognitive ability, such as variation in effectiveness and efficiency of within-occasion learning. Understanding these aspects require focusing on the analysis of performance trajectories and change points (Figure 3, points 7–9). When we ask whether and how such within-individual parameters differ between individuals, we open the potential for more holistic models of cognitive ability and cognitive performance differences under more typical everyday conditions.

#### 3.4.3. Variability in Performance vs. Response Time

The choice of dependent variable in cognitive ESM studies is a further consideration. The preceding discussion assumes accuracy/error scores are the dependent variable; however, another candidate could be response time, or some function of it. Understanding within-individual variation in personality does not necessitate the analysis of response time, as they are not likely to tell us much about the dynamic nature of participants’ personality variation beyond, say, identifying non-serious responding; however, response time is often important for understanding cognitive performance even on tasks where the focus is on accuracy. A critical aspect of typical cognitive performance is motivation and engagement; it is not enough to just finish the task, the participant must actively engage with it, particularly if it is a dynamic one (Goff and Ackerman 1992; von Stumm et al. 2011). Analysing structured variability in response time data alone and in conjunction with accuracy data may provide insights into strategy use, cognitive exploration, and flexibility, as well as task engagement.

#### 3.4.4. Reliability and Boundary Effects

Well-established methods exist for gauging the reliability of personality and cognitive measures. They rely on standardised administration as well as statistical assumptions, such as local independence (Birney et al. 2022); however, applications of standard reliability coefficients are challenging when applied to data from dynamic cognitive tasks and repeated measures, which entail within- and between-person variation in structural features, such as administration under different situations and when differential feedback is provided.

A potential solution lies in the fact that repeated measures data lends itself to multilevel modelling (MLM) and the decomposition of variance into structured effects both between- and within-individuals. MLM allows for the estimation of random effects, which are model-implied individual parameter estimates within and across levels of the data. These have promise for use as adjusted predictors of other outcomes of interest. Liu et al. (2021) examined the reliability of MLM random effects using both simulated and empirical data and multilevel regression and structural equation models. They concluded that empirical Bayes estimated random effects produce biased regression coefficients but that a wide variety of factors influence their reliability: larger variance in Level 1 predictors, larger variance in Level 2 random effects, and a higher number of observations per person each increase reliability, whereas larger Level 1 residual variance decreases reliability. As a caveat to these findings, they reported that individual-specific differences in these factors further influence the reliability of random effects, e.g., random effects of individuals with a low number of observations are likely to be biased towards zero. Accordingly, they caution researchers to understand why data is missing before using random effects as predictors, e.g., individuals may consistently miss response occasions for a reason that is relevant to the variables of interest, thus not providing an accurate picture of within-person factors.

Depending on the parameters extracted from the data, ESM studies may also yield boundary effects and heteroscedasticity. This is particularly likely if the dependent variable under investigation is an indicator of within-person variability. People whose performance is consistently at the higher or lower end of a trait dimension will by definition have less space for their states to vary in the scale-bounded direction. For example, those both low and high in conscientiousness or abstract reasoning capacity tend to vary less. Methods for adjusting for such boundary effects exist (Mestdagh et al. 2018) and have been successfully applied in personality data (Beckmann et al. 2020). They could be leveraged when analysing within-person cognitive data also.

## 4. Part 3: A Case Study and Initial Findings

We have recently run two studies in our lab that provide early insights into feasibility of the ESM paradigm to capture within-individual variability in cognitive performance. In the first study, 101 undergraduate university student participants were sent 12 dynamic microworld-style tasks over a three-week period. Tasks were emailed to participants on weekdays between 10 a.m. and 2 p.m., and participants were told they had six hours to complete each task (although, in practice, where participants contacted us after the deadline, we provided them with a chance to make up the ‘missed’ task). This time period and six-hour window was selected to mirror the usual working time of a university student, and was based on a judgment of the commitment university student participants would be likely to meet. The average number of tasks completed was 9.18 tasks (SD = 3.47 tasks) and the median was 10 tasks, with an overall task response rate of 76.5% across participants. Across the completed tasks, the average delay between receiving and completing the task was 3.43 h (SD = 9.93 h) and the median delay was 0.99 h. The number of tasks completed and the average delay between receiving and completed tasks was significantly correlated such that the higher the number of tasks completed, the shorter the delay between receiving and completing a task (*r* = −0.251, *p* < .05). In total, 72.4% of tasks sent were completed within the allotted six-hour time window.

In the second study, 89 undergraduate university student participants were sent five reasoning-style tasks over a two-week period and completed a longer single session reasoning test. One task was emailed to participants every weekday between 10 a.m. and 2 p.m. for two weeks until participants had completed all five tasks, or the two weeks was over, whichever came first. Participants were told they had 24 h to complete each task; this time window was extended based on qualitative feedback from Study 1 participants and to examine whether extending the time window affected response rates and rapidity of completion. The average number of tasks completed was 4.02 tasks (SD = 1.77 tasks) with a median of 5 tasks, with an overall task response rate of 80.4% (marginally higher than Study 1). All five tasks were completed by 71.91% of participants. The average delay between receiving and completing the task was 6.24 h (SD = 4.43 h) and the median delay was 5.49 h. Unlike Study 1, there was no correlation between the number of tasks completed and the average delay between receiving and completing the task (*r* = −0.024, *p* > .05). In total, 80.4% of tasks were completed within the allotted 24 h time window, which is marginally lower than Study 1.

Typical response rates in ESM studies lie between 70–90% (Fisher and To 2012). Csikszentmihalyi and Larson (2014) report response rates of 70% in high school students, 73% in blue collar workers, 85% in clerical workers, and 92% in managerial workers. Our respective response rates of 76.5% and 80.4% are promising indicators that, when short-term repeated measures cognitive assessments are run in a way that is minimally demanding, response rates are similar to those found in more frequent ESM personality assessments, at least in a university student sample.

## 5. Conclusions

While there is empirical evidence for within-individual variability in cognitive task performance, our theories of and methods for understanding cognitive ability have not accounted for this. Our typical cognitive performance in education and work is inherently variable; over time, we perform differently in response to changes in the situation and tasks we are presented with, and differences in person factors, such as personality motivational traits, and task learning and strategy use; however, the study of intelligence continues to rely on between-person comparisons of single-occasion test scores that do not consider the possibility or implications of within-individual variability in *cognitive ability* impacting *cognitive performance*. Furthermore, there have been few attempts made to understand the extent, magnitude, and structure of this variability. Without understanding this, our theories of cognitive ability will remain incomplete.

In this paper, we presented a case for why we should study within-individual variability in cognitive ability. In doing so, we highlighted the utility of a within-individual approach in related fields, particularly the personality literature. In personality research, studying within-individual variability in personality expression has fundamentally altered our understanding of personality as a construct by facilitating a within-person process account of structured personality variation over time. These findings have been unearthed by pivoting away from between-individual single-occasion administration and analysis methods towards within-individual methods. We believe that there are lessons to be learned from this approach for those who research cognitive ability. Specifically, we advocate for the use of the ESM to generate rich, within-person data; however, we cannot simply copy and paste the methods used in personality research as there are important distinctions to be made between the theory, methods, and analytical approaches used.

Within-individual variability in cognitive performance is multifaceted and complex, possessing both between-occasion and within-occasion variability components and likely to be influenced by a broad range of situation, task, and person factors. Conversely, within-individual variability in personality represents relatively transient momentary fluctuations in personality states in response to situational changes that can be captured with single- or few-item assessments. Consequently, significant consideration must be given to the design of cognitive ESM studies and the analysis of resulting data. We suggest that dynamic tasks will be required to capture between- and within-occasion performance variation. Moreover, researchers should seek to experimentally manipulate various situation, task, and person factors to elucidate their unique and interactive contributions to between-person differences in within-individual variability. As a result, cognitive ESM studies are likely to be longer and more demanding than their corresponding personality assessments, which has implications for the spacing of assessments and participant recruitment and retention strategies. Moreover, researchers will need to carefully consider the phenomena of interest (i.e., within-individual cognitive performance variability) when deciding which performance parameters to extract from the data and when making data cleaning decisions.

Initial findings from our lab suggest that ESM assessments are feasible, with similar response rates to those in the personality literature when the assessments are of longer duration and spacing. Our future research will focus on the factors influencing participation in this style of assessment, how we can extract parameters from this data, and eventually the incremental predictive utility of these parameters (over and above between-person performance indices) for assessing success in educational and workplace environments. In presenting these arguments, we hope that researchers will be prompted to consider how integrating a within-person perspective into cognitive ability could benefit our theories and methods for assessing cognitive ability in the 21st century.

## Data Availability

Data available upon request due to restrictions.

## Notes

1 By change based on past responses, we do not mean adaptive psychometric testing, but rather a more qualitative change in item type.

2 ESM is also known as ecological momentary assessment (EMA) and ambulatory assessment.

3 Task design is related, in part, to the particular ability being assessed. For instance, Gf, Gc, and working-memory should each entail task designs that capture their respective within-person processes.
